# Sole trisomy 6 an uncommon finding in pediatric acute myeloid leukemia, probably associated to bad prognosis

**DOI:** 10.1186/s13039-020-00509-y

**Published:** 2020-09-04

**Authors:** Sinhue Alejandro Brukman-Jimenez, Lucina Bobadilla-Morales, Jorge Román Corona-Rivera, Pablo Alejandro Chávez-Panduro, Citlalli Ortega-de-la-Torre, Uriel Francisco Santana-Bejarano, Elizabeth Torres-Anguiano, Lucero Mendoza-Maldonado, Fernando Antonio Sánchez-Zubieta, Alfredo Corona-Rivera

**Affiliations:** 1 Unidad de Citogenética, Servicio de Hematología y Oncología Pediátrica, División de Pediatría, Nuevo Hospital Civil de Guadalajara, “Dr. Juan I. Menchaca”, Guadalajara, Jalisco Mexico; 2 Instituto de Genética Humana “Dr. Enrique Corona Rivera”, Laboratorio de Citogenética y Genómica, Centro Universitario de Ciencias de la Salud, Universidad de Guadalajara, Sierra Mojada Street #950, CP-44340 Guadalajara, Jalisco Mexico; 3 Servicio de Hematología y Oncología Pediátrica, División de Pediatría, Nuevo Hospital Civil de Guadalajara, “Dr. Juan I. Menchaca”, Guadalajara, Jalisco Mexico

**Keywords:** Sole trisomy 6, Acute myeloid leukemia, Overall survival

## Abstract

**Background:**

Acute leukemias represent the main malignancies occurring among children under the age of 15 years. Around 17% corresponds to acute myeloid leukemia (AML). The cytogenetic analysis of bone marrow complements the diagnosis of hematological malignancies, therefore finding chromosomal aberrations provides a more reliable prognosis of the disease. Among the cytogenetic aberrations, sole trisomy is frequent in malignant neoplasias, but few cases related to AML have been reported.

**Case presentation:**

We report a sole trisomy 6 in a pediatric patient diagnosed as AML M4 and poor progression. We carried out a literature review of AML patients with sole trisomy 6 and compared their evolution against AML patients with normal karyotype.

**Conclusions:**

This is the first case of pediatric AML M4 with this cytogenetic finding. Sole trisomy 6 is infrequently reported in AML but scarce in pediatric cases. Based on overall survival analysis, we suggest that sole trisomy 6 could be associated with poor prognosis, in both, adult as well as pediatric AML.

## Background

Leukemia is the most frequent malignant neoplasia among the pediatric population. Acute leukemias represent 32% of malignancies occurring in children under the age of 15 years and around 17% of them are due to acute myeloid leukemia (AML) [1]. AML includes a genetically and clinically heterogeneous group classified by cytogenetic, immunophenotypic, and molecular findings in the patient [2]. Trisomy of chromosome 6 as a sole cytogenetic abnormality has been observed in malignant neoplasias [3, 4]. In the case of trisomies of AML, + 8 is the most frequently observed, followed by trisomy 4, 9, 11, 13 and 21 [5], but trisomy 6 is infrequent [3, 4]. We scored 29 reported cases, 24 of them adults, and only 5 that corresponded to pediatric cases. The presence of AML-M4 cases was scarce in adults and not observed in pediatric cases. Prognosis of these cases has not been clarified [4]. We present a pediatric patient with AML-M4 and trisomy 6 as a sole cytogenetic abnormality confirmed by classical cytogenetics and molecular methods, literature update and prognostic implications.

## Case report/case presentation

**Fig. 1:**
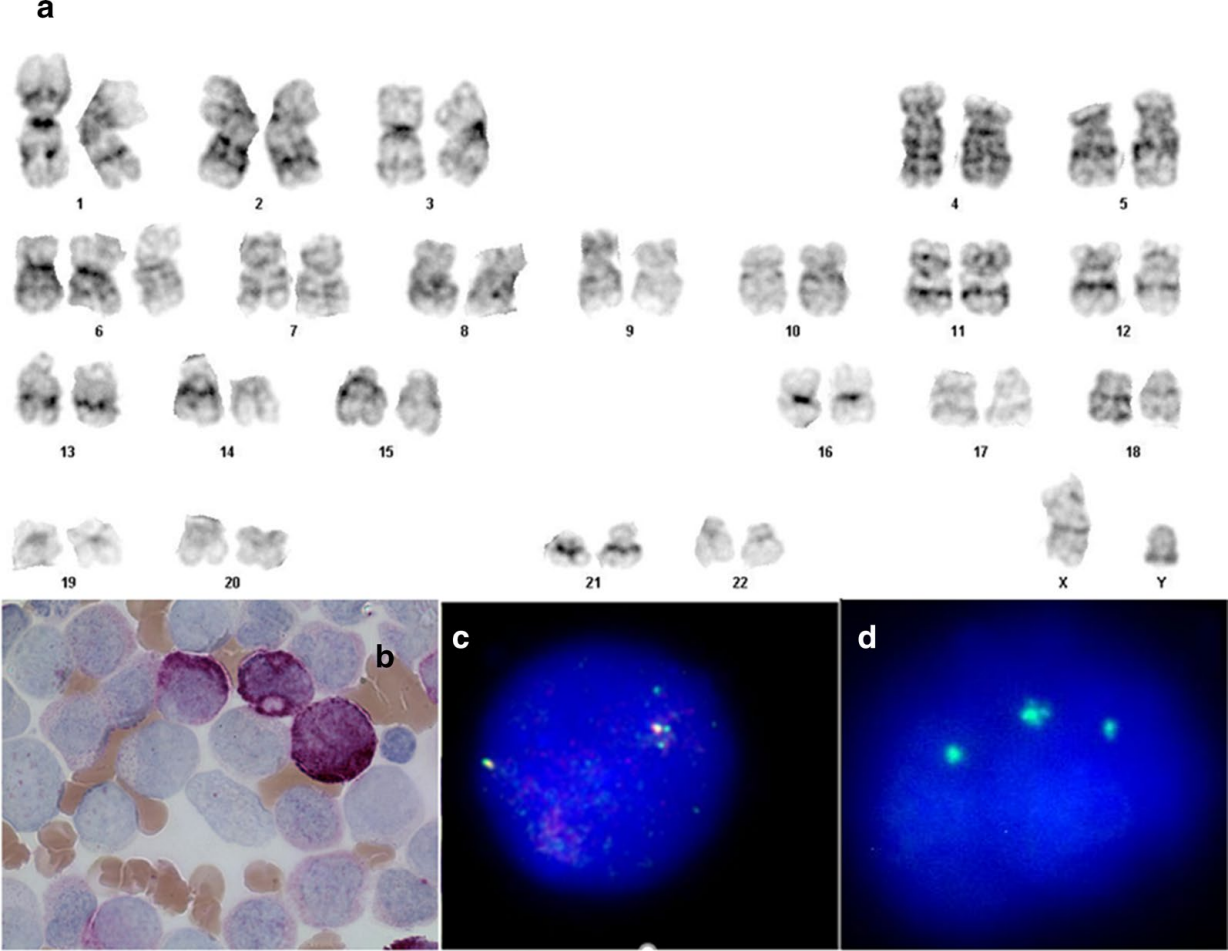
**a** Representative image of pure trisomy 6 with GTW banding (350 band resolution) found in bone marrow sample. **b** Sample morphology obtained by bone marrow aspirate with the presence of monocytes and myelomonocytes compatible with the diagnosis of LMA-M4 by FAB classification. **c** FISH for the LSI CBFB dual-color break apart Rearrangement Probe, where one fusion was found. This indicates that to undisrupted CBFB genes were present. **d** FISH for the Vysis CEP 6 centromere probe (D6Z1), shows 3 signals in an interphase cell

A 14-year-old male patient, he was the third child of non-consanguineous healthy parents without carcinogens exposition reported, from a dizygotic pregnancy, delivered by C-section, weight: 3350 g, stature 52 cm, with no otherwise pathological background, and not reported exposure to carcinogens, was admitted in 2016 with 8-day of cervical lymphadenopathy, intermittent fever, polydipsia, emesis, and hyporexia. Physical examination at the moment of admission, he showed mucotegumentary paleness, gingival hypertrophy, and palpable bilateral cervical lymph nodes of 15 mm, as well as right supraclavicular palpable 15 mm lymph node; palpable hepatic flange at 2 cm below the costal ridge, not palpable spleen. Upon admission, his blood counts were: Hemoglobin: 5.7 g/dL, WBC: 244,000/μL, Platelets: 52,000/μL. The bone marrow aspirate showed hypercellularity, absent red and megakaryocytic series, increased white series with a predominance of monocytic series, and myeloblasts (Fig. 1b). By cytochemistry, myeloperoxidase positive blasts we reported. Immunophenotype reported a myeloid population of 72%, expressing CD13+, CD33+, HLA-DR+, CD38+, CD117+, and a second monocytic population of 20%, expressing CD14+, CD33+, CD36+, CD64+, CD38 and CD45+. All the above data were compatible with Acute Myelomonocytic Leukemia or French–American–British (FAB) M4 classification [2]. He started chemotherapy, receiving cytarabine (100 mg/m^2^/dose q12h), daunorubicin (50 mg/m^2^/dose q48h) and etoposide (100 mg/m^2^/dose q24h). On day 2, an increase in the leukocyte count was reported: 500,000/μL with a monocytes predominance of 75%; leukophoresis was indicated, and cytarabine induction was initiated. He died on day 3, with hyperleukocytosis of 728,000 μ/L, acute renal failure, and pulmonary leukostasis. In this study patient data were protected and maintained as anonymous. The parents gave their written informed consent to use clinical data.

### Chromosome and fluorescence in situ hybridization analysis

The bone marrow sample was obtained for cytogenetic and molecular diagnosis. Chromosomal preparations were obtained from cell cultures of 0 and 24 h of incubation cultured with RPMI, supplemented with l-glutamine, fetal bovine serum, and antibiotic. Chromosome banding was performed with trypsin and Wright solution. For the molecular diagnosis of trisomy 6, FISH was performed with the Vysis CEP 6 probe (D6Z1). Besides, Fluorescence In Situ Hybridization was performed for CBFB gene disruption from 16q22 chromosomal rearrangement with the LSI CBFB Dual Color Break Apart Probe (Vysis, cat. 05J65-001 (Fig. 1c). The finding of one red signal, one green signal, and one fusion is a representative finding of an abnormal chromosomal rearrangement, in this case, 2 fusions were found, it indicates a normal result, supporting the finding of sole trisomy 6.

### Molecular diagnosis

qPCR was performed to the search of inv(16)(p13.1q22) by CBFB-MYH11 fusion transcripts from cDNA using applied biosystems ™ high capacity cDNA reverse transcription kit and Thermo Fisher Scientific *CBFB*-*MYH11* fusion transcripts panel kit. The FLT3/ITD amplification was performed on a Proflex 96-well PCR system (Applied Biosystems) thermocycler using 11F (5′-6FAM-CAA TTT AGG TAT GAA AGC C-3′) and 12R (5′-GTA CCT TTC AGC ATT TTG AC-3′) PCR primers. Capillary electrophoresis separations were performed using a 3130 Genetic Analyzer (Applied Biosystems) and GeneScan-500 LIZ size standard. The fragment analysis was carried out with Gene-Mapper v.3.5 software (Applied Biosystems).

To corroborate the absence of fusion genes involved in leukemia chromosomal rearrangements, we performed a nested multiplex RT-PCR (HemaVision^®^-HV01-28 N, DNA Technology A/S) to the search of 28 common AML and ALL fusion transcripts. The procedure was described elsewhere [6]. The evaluated 28 fusion transcripts were: del1(p32) (STIL-TAL1), t(1;11) (p32;q23)(MLL-EPS15), t(1;11)(q21;q23) (MLL-MLLT11), t(1;19)(q23;p13) (TCF3-PBX1), t(3;5)(q25;q34) (NPM1-MLF1), t(3;21)(q26;q22) (RUNX1-MDS1/EVI1), t(4;11)(q21;q23) (MLL-AFF1), t(5;12)(q33;p13) (ETV6-PDGFRB), t(5;17)(q35;q21) (NPM1-RARA), t(6;9(p23;q34) (DEKNUP214), t(6;11)(q27;q23) (MLL-MLLT4), t(8;21)(q22;q22)(RUNX1-RUNX1T1), t(9;9)(q34;q34) (SET-NUP214), t(9;11)(p22;q23) (MLL-MLLT3), t(9;12)(q34;p13) (ETV6-ABL1), t(9;22)(q34;q11) (BCR-ABL1), t(10;11)(p12;q23) (MLL-MLLT10), t(11;17)(q23;q21) (MLL-MLLT6), t(11;17)(q23;q21) (ZBTB16-RARA), t(11;19)(q23;p13.1) (MLL-ELL), t(11;19)(q23;p13.3) (MLL-MLLT1), t(12;21)(p13;q22) (ETV6-RUNX1), t(12;22)(p13;q11) (ETV6-MN1), t(15;17)(q24;q21) (PML-RARA), inv(16)(p13;q22) (CBFB-MYH11), t(16;21)(p11;q22) (FUS-ERG), t(17;19)(q22;p13) (TCF3-HLF), t(X;11)(q13;q23)(MLL-FOXO4). Any fusion genes were found in this probe.

### Statistical analysis

Overall survival (OS) is defined as the time from the day of diagnosis to death or last visit [7]. Overall survival was calculated by methods of Kaplan & Meier and compared between groups with the log-rank test [8]. The analyses were performed using SPSS software version 22 (SPSS, Chicago, IL, USA).

### Database research

To establish the relationship found between the cytogenetic report (sole trisomy 6) and bone marrow morphology (AML), the search for previous reports with these same variants was carried out in Mitelman Database of Chromosome Aberrations and Gene Fusions in Cancer [9] (http://cgap.nci.nih.gov/Chromosomes/Mitelman), and the NCBI database (http://www.ncbi.nlm.nih.gov/pubmed) with the combination of the terms “Trisomy 6”, “AML”, and “M4”. We found 29 previous reports of trisomy 6 and the morphological diagnosis of AML: 24 cases corresponded to adults and only 5 to pediatric patients (1 FAB M3, 1 M7 and 3 unclassified (Table 1).

**Table 1:** Cases of acute myeloid leukemia with sole trisomy 6 and their laboratory findings

Case	Diagnosis	Karyotype	Survival (months)	Age (years)	Sex	WBC*	Hb**	PLT***	References	Country
*Adult cases*										
1	AML-NS	NA	36	74	M	NA	NA	NA	Panani et al. [20]	Greece
2	AML-M4	47,XX, + 6[8/18]	1	50	F	NA	NA	NA	Testa et al. [21]	USA
3	AML-M5	47,XX,+6[58/58]	4	24	F	NA	NA	NA	Weh et al. [22]	Germany
4	AML-M2	47,XX,+6[2/7]	NA	81	M	NA	NA	NA	Chan et al. [23]	Hong Kong
5	AML-M2	47,XX,+6[3/8]	NA	NA	F	NA	NA	NA	Berger et al. [24]	France
6	AML (M2)	47,XY,+6[15/21]	> 1	55	M	NA	NA	NA	UKCCG [25]	U.K.
7	AML (M5a)	47,XX,+6[17/17]	> 15	55	M	NA	NA	NA	UKCCG [25]	U.K.
8	AML-NS	47,XX,+6[2/?]	22	37	F	NA	NA	NA	UKCCG [25]	U.K.
9	AML-M2	47,XX,+6[3/9]/46,XX[6/)9]	3	63	F	4.8	13.5	6	Jonveaux et al. [18]	France
10	AML-M2	47,XX,+6[17/19]/46,XX[2/19]	1+	28	F	4	11	53	Jonveaux et al. [18]	France
11	AML-M4	47,XX,+6[19/30]/46,XX[11/30]	22	66	F	2.6	8.1	51	Mohamed et al. [11]	USA
12	AML-M1	47,XX,+6[30/30]	1	74	M	29.4	9.3	26	Mohamed et al. [11]	USA
13	AML-M1	47,XY,+6[20/20]	4	22	M	4.4	12.5	238	Mohamed et al. [11]	USA
14	AML-M1	47,XY,+6[7/20]/46,XY[13/20]	10	40	M	86.5	11.1	63	Mohamed et al. [11]	USA
15	AML-MRC	47,XX,+6[39/40]/46,XX[1/40]	> 47	37	F	5.1	12.2	11	Mohamed et al. [11]	USA
16	AML-NS	47,XX, +6[8/23]/46,XX[15/23]	27	41	F	NA	NA	NA	De Souza et al. [26]	Brazil
17	AML-NS	47,XX,+6	NA	NA	F	NA	NA	NA	Kerndrup and Kjeldsen [27]	Denmark
18	AML-NS	47,XX,+6 [11]	NA	61	F	NA	NA	NA	Beyer et al. [28]	Switzerland
19	AML-M1	47,XX,+6[20]	23	51	M	20.77	13.5	4	Yu et al. [4]	South Korea
20	AML-M2	47,XX,+6[20]	> 58	25	F	82.94	8	109	Yu et al. [4]	South Korea
21	AML-M4	47,XX,+6[4/20]/46,XX,[16/20]	3	82	M	5.08	6.4	21	Yu et al. [4]	South Korea
22	AML-M7	47,XX,+6[12/20]/46,XX[8/20]	**1**	21	F	56.3	5.4	85	Gupta et al. [10]	India
23	AML-NS	47,XX,+6	1	50	F	NR	8.5	11	Aydin et al. [29]	Turkey
24	AML-M5	47,XY,+6	3	75	M	103.8	7.3	80	Manabe et al. [12]	Japan
*Pediatric cases*										
25	AML-NS	47,XY,+6	NA	11	M	NA	NA	NA	Philip et al. [13]	Denmark
26	AML-NS	47,XX,+6[8]	27	8	F	99	NA	NA	Benedict et al. [14]	USA
27	AML-(M3)	47,XY,+6	> 7	8	M	NA	NA	NA	UKCCG [25]	U.K.
28	AML-M7	47,XX,+6[9/20]/46,XX[11/20]	NA	1	M	16.4	7.6	6	McCullough et al. [15]	Ireland
29	AML-NS	47,XX,+6	18	8	F	NA	NA	NA	Koh et al. [19]	South Korea
30	AML-M4	47,XX,+6[12/15]/46,XX[3/15]	1	14	M	244	5.7	52	Present report	

WBC *: White blood cells measured in 10^9^/L; Hb **: Hemoglobin measured in g/dL; PLT ***: Platelets measured in 10^9^/L; AML-NS: Acute Myeloid Leukemia non otherwise specified by FAB classification; NA: Not available data. U.K.: United Kingdom; USA: United States of America

## Results

The observed karyotype from bone marrow aspirate was: 47,XX, +6[12/15]/46,XX[3/15]. FISH studies revealed the presence of additional chromosome 6 centromere repetitive sequences signal in 143 out 200 cells: nuc ish(D6Z1)x3[143/200]/(D6Z1)x2[57/200], and the absence of 16q22 chromosome rearrangements (Fig. 1a, c, d).

qPCR studies: CBFB-MYH11 fusion transcripts expression was not observed, in consequence, we concluded that inv16(p13.1q22) was not present. The FLT3/ITD amplification mutation was positive.

Nested multiplex PCR assay to detect 28 common fusion transcripts in AML and ALL was reported negative. This supports the absence of the most common chromosomal rearrangements in AML in our patient.

In Table 1, the reported cases with trisomy 6 as the sole chromosomal abnormality are summarized. 24 adults and 5 pediatric cases were scored.

**Fig. 2:**
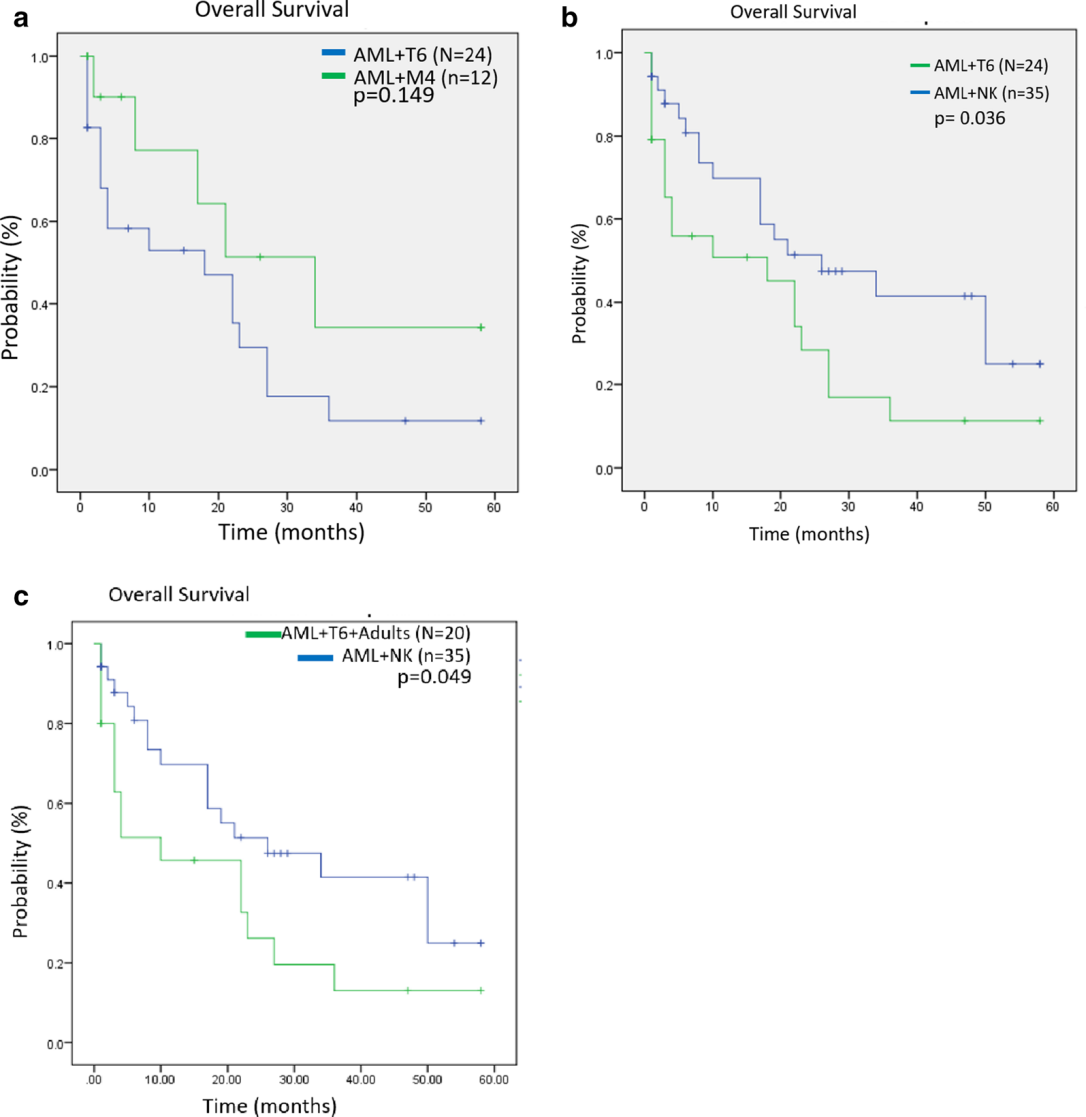
Overall survival (OS) by karyotype condition calculated by Mantel-Cox test. **a** OS of patients previously reported with AML and trisomy 6 (AML + T6) compared to childhood patients from our hospital with AML M4 and normal karyotype (AML-M4 + NK). **b** OS of patients with AML + T6 compared to childhood patients from our hospital with AML and normal karyotype (AML + NK). **c** OS of patients with AML + T6 adults (n = 20) previously reported compared to childhood patients from our hospital with AML and normal karyotype (AML + NK)

To search the prognosis, we performed an OS analysis. In this context, Yu et al. [4], did not identify trisomy 6 as a prognosis marker. It also had been discussed that more studies were needed to elucidate the role of trisomy 6 in prognosis [10]. Due to there are no reports of trisomy 6 in LMA M4 pediatric population we tried to define if trisomy 6 could be a marker of poor prognosis. To determine the probable prognostic impact of this cytogenetic finding, we compared the available OS obtained from previous cases reported with sole trisomy 6 and AML patients (AML + T6, n = 24), including our case report, versus pediatric patients from our hospital with LMA M4 and normal karyotype (NK) (n = 12) (Table 1). The median OS in AML + T6 was 18.6 months compared to 31.8 months in LMA-M4 + NK. Differences between groups was calculated by mantel-cox test, and it was not significant (p = 0.149) (Fig. 2a). AML + T6 group was also compared to pediatric patients from our hospital with different types of AML + NK (n = 35, FAB classification: M1 = 2, M2 = 6, M3 = 5, M4 = 13, M5 = 7, M6 = 1, M7 = 1, OS 1 to 58 months). In AML + T6, the median OS was 17.9 months compared to 30.6 months in AML + NK, which were significant by mantel-cox test of p = 0.039 (Fig. 2b). Furthermore, we compared adult patients from Table 1 (n = 20) versus pediatric AML + NK group observing a significant difference (mantel-cox test, p = 0.049) (Fig. 2c); OS of patients with AML + 6 childhood (n = 4) previously reported compared to childhood patients from our hospital with AML and normal karyotype (AML + NK) were not significant.

## Conclusion

In our review, twenty-nine AML cases with trisomy 6 as a sole cytogenetic aberration (Table 1) had been previously reported. Most of the cases were adults (24/29, age range 21–82 years old) and only a 6 pediatrics (age range 1–14 years old) including the present case. There was no predominance of sex. Ten of them were diagnosed as AML but no FAB classification was specified, four were FAB classified as AML-M1, one as AML-MRC, five as AML-M2, one as AML-M3, four as AML-M4, three as AML-M5 and two as AML-M7. With this variety of e, we cannot infer a possible association of morphology FAB classification and trisomy 6 as seen previously [4, 11, 12]. As the few reported cases showed, trisomy 6 is presented predominantly in adults. Our case is the first pediatric (14 years old) with AML-M4. Interestingly, in all AML-M4 cases including the present case, a proportion of normal cells without +6 was observed, and the proportion of trisomy 6 cells varied from 20 to 80%. Additionally, it is to be noted that among them, the two patients with the highest percentage of trisomy 6 cells had the lowest chances of survival. Although this could suggest a prognostic factor of this karyotype in AML-M4, nevertheless, more cases are needed to elucidate this topic.

In the previous sole trisomy 6 cases, only 3 patients were reported with AML M4, and it was presented in adults 2 of the reports refer to the cytogenetic finding as a poor prognosis factor when having a survival factor of 1–3 months. To date, few cases have been reported with this chromosomal complement in the presence of AML and there is no case of trisomy 6 in the pediatric population in the presence of AML M4. Evidence in our case comes from centromere FISH studies, that exhibit 3 centromere sequence 6 chromosome signals. Additionally, no other alteration was found in the 28 genes analyzed by nested multiplex PCR. Also, we ruled out the presence of FAB condition Ma eo, because CBFB FISH exhibited a normal pattern. Previous pediatric reported cases were unable to perform these analyses [13, 14], just one case [15], used WCP 6 chromosome to confirm the presence of +6, to complement the cytogenetical finding. It seems that the sole +6 condition yield to a particular myeloid entity.

Yu et al. [4], concluded that more studies are needed to establish the clonality of AML with + 6. To our knowledge, there have been no reports with additional molecular studies like ours. *FLT3* (the Fms-like tyrosine kinase 3) gene encodes a receptor that supports the survival, proliferation, and differentiation of hematopoietic progenitor cells in the bone marrow. In patients with a diagnosis of AML may behave mutations in *FLT3* gene. Two kinds of mutations are more frequent: internal tandem duplications (ITDs) of juxtamembrane domain and missense point mutations, in the tyrosine kinase domain [16]. ITDs are associated with poor prognosis [17]. Our patient was reported as *FLT3* positive, giving a poor prognosis joined with the age at the diagnosis moment probably gives a fatal outcome. It is too early to suggest a possible association between sole trisomy 6 in AML and *FLT3* ITDs, additional molecular studies are required to investigate the *FLT3* status in these patients, but in our patient, this could be associated to bad prognostic.

Pure trisomy 6 is a chromosomal abnormality rarely reported as a cytogenetic finding in malignant hematologic neoplasms [15]. It has been suggested that this chromosomal rearrangement could belong to a poor prognosis subgroup related to MDS and associated with cytopenia and bone marrow hypoplasia [11]. Our statistical evaluation using all de available OS previously reported in AML patients with sole trisomy 6 against a comparative group of patients from our hospital with different types of AML but normal karyotype, contribute to the hypothesis previously suggested that trisomy 6 in AML could be related to bad prognosis in patients with this unusual cytogenetic finding.

The effect of an extra copy of a complete chromosome on the development of leukemogenesis is unknown, but some theories could explain its effects. The first is the direct effect of gene dosage generating an overexpression, and the second is the presence of cryptic gene rearrangements or gene mutations in the extra chromosome [15]. Following the suggestion of this theory, the search for multiple nested RT-PCR fusion genes, HemaVision^®^ kit, was performed to search the 28 most common fusion genes, which was reported as negative. On the other hand, this clonal cytogenetic abnormality is reported with poor treatment response and poor survival [18], probably linked with the primitive nature of blasts [11]. Our patient wasn’t positive to HLA-DR and CD34 as other reports [10, 30]. In a pediatric AML case study by microarrays including a case with trisomy 6, Koh et al. [19], proposed than a poor prognosis related to this aneuploidy in AML could be due to candidate *IRF4* (6p25.3) and *DEK* (6q22.3) genes associated to leukemogenesis.

In summary, sole + 6 condition may yield to a particular myeloid entity. Sole trisomy 6 has been reported in multiple hematologic alterations, such as aplastic anemia and myeloproliferative syndromes, but in a few cases of primary acute myeloid leukemia, and this is the first case of reported infantile AML M4. Due to the evolution described in this case and survival reported in patients with sole trisomy 6 against normal karyotype, the finding of pure trisomy 6 could be probably attributed to poor prognosis in AML, in both, adults and children. Further studies will elucidate the clinical effect of an extra chromosome 6 in AML and its significance.

## Data Availability

The datasets generated and/or analyzed during the current study are not publicly available due because the data of the patients treated at the reference institution, are only available for use within the institution, but are available from the corresponding author on reasonable request.

## Abbreviations

AML: acute myeloid leukemia
RT-PCR: reverse transcription polymerase chain reaction
Hb: hemoglobin measured in g/dL
OS: overall survival
ITD’s: internal tandem duplications
FAB: the French–American–British
AML-M4: acute myeloid leukemia M4 by FAB
WBC: white blood cells measured in 10^9^/L
LOH: loss of heterozygosity
AML + T6: acute myeloid leukemia and trisomy 6
FLT-3: the Fms-like tyrosine kinase 3
